# A clinical diagnosis of oral leukoplakia; A guide for dentists

**DOI:** 10.4317/medoral.22292

**Published:** 2017-12-24

**Authors:** Vinicius C. Carrard, Isaäc van der Waal

**Affiliations:** 1 Federal University of Rio Grande do Sul, School of Dentistry, Porte Alegre, Rio Grande do Sul, Brazil; 2 VU medical center/ ACTA, Dept. of Oral and Maxillofacial Surgery/Pathology, Amsterdam, the Netherlands

## Abstract

**Background:**

In view of the many white or predominantly white lesions of the oral mucosa it is a challenge for dentists to clinically identify a leukoplakia, being a potentially (pre)malignant lesion.

**Material and Methods:**

Based on the available literature and experience of the authors the parameters of a clinical diagnosis of oral leukoplakia have been studied.

**Results:**

A guide has been presented that should help dentists to establish a clinical diagnosis of leukoplakia as accurate as possible.

**Conclusions:**

Probably in most parts of the world dentists will need the help of a specialist for confirmation or exclusion of the clinical diagnosis of oral leukoplakia and for further management of the patient, including patient information.

** Key words:**Oral diseases, oral leukoplakia.

## Introduction

Oral leukoplakia, being a predominantly white change of the oral mucosa, is the most common potentially (pre)malignant lesion. It is a relatively rare disease with an estimated prevalence of less than 1%. Men and women are more or less equally affected. Oral leukoplakia rarely occurs in the first two decades of life and is much more common in tobacco users than in non-tobacco users. Leukoplakia may occur everywhere in the oral cavity and is often asymptomatic otherwise. The clinical diagnosis is primarily based on visual inspection and manual palpation. There are no other useful diagnostic aids for the clinical diagnosis.

The histopathological findings in leukoplakia range from hyperkeratosis without epithelial dysplasia to various degrees of epithelial dysplasia and even carcinoma in situ, frank squamous cell carcinoma and verrucous carcinoma.

The annual risk of malignant transformation of leukoplakia, if not malignant already at the first visit, is approximately 2%-3%. There are many, statistically somewhat useful, predictive factors of malignant transformation, such as the seize of the lesion, the clinical subtype, the oral subsite, and the presence or absence of epithelial dysplasia as assessed by histopathological examination, but these can not reliably be used in the individual patient. This also applies to numerous molecular markers that have been reported as predictive markers of malignant transformation.

Spontaneous regression of leukoplakia is exceedingly rare. Surgical and non-surgical treatments have not been shown to be effective in preventing possible future malignant transformation (1).

It is a challenge for the general dentist to be able to timely identify a leukoplakia. The most recent definition, formulated at a World Health Organization supported meeting, reads: “A white plaque of questionable risk having excluded (other) known diseases or disorders that carry no increased risk for cancer” (2). For use by clinicians this definition may be modified into : “A predominantly white, non-wipable lesion of the oral mucosa having excluded clinically, histopathologically or by the use of other diagnostic aids other, well-defined predominantly white lesions”. In fact, a diagnosis of leukoplakia is one by exclusion of a large number of well-defined common and also uncommon lesions and disorders that may occur in the oral mucosa. A dentist-general practitioner can not be supposed to be familiar with all such lesions and disorders. The present text is intended to serve as a guide for use in the daily practise.

## A clinical diagnosis of leukoplakia

A clinical diagnosis or differential diagnosis of a mucosal lesion is the result of a number of parameters. The importance of each parameter varies according to the type of lesion. The parameters and their relevance with regard to the establishment of a clinical diagnosis of leukoplakia have been listed in  Table 1.

**Table 1:**
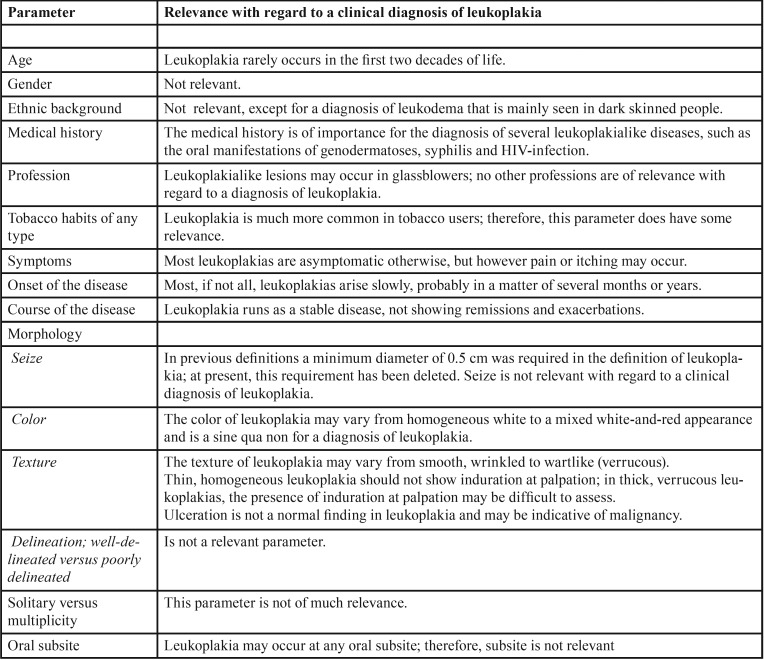
Parameters and their relevance with regard to the process of establishing a clinical diagnosis of oral leukoplakia.

Traditionally, leukoplakias are clinically subdivided in a homogeneous and a non-homogeneous variant. In homogeneous leukoplakia the lesion is uniformly white and the surface is flat or slightly wrinkled. In non-homogeneous leukoplakia there is a mixed white-and-red color (“erythroleukoplakia”); the surface may be flat, speckled or nodular. A separate variant of non-homogeneous leukoplakia is the wartlike, verrucous type. In case of a widespread presentation and depending on other criteria such as worsening along time, this type is referred to as proliferative verrucous leukoplakia. Non-homogeneous leukoplakias carry, statistically, a higher risk of malignant transformation.

A list of leukoplakialike, well-defined predominantly white lesions and their main diagnostic criteria is depicted in  Table 2, Table 2 continue. This table may be helpful as a checklist in distinguishing such entities from leukoplakia.

**Table 2:**
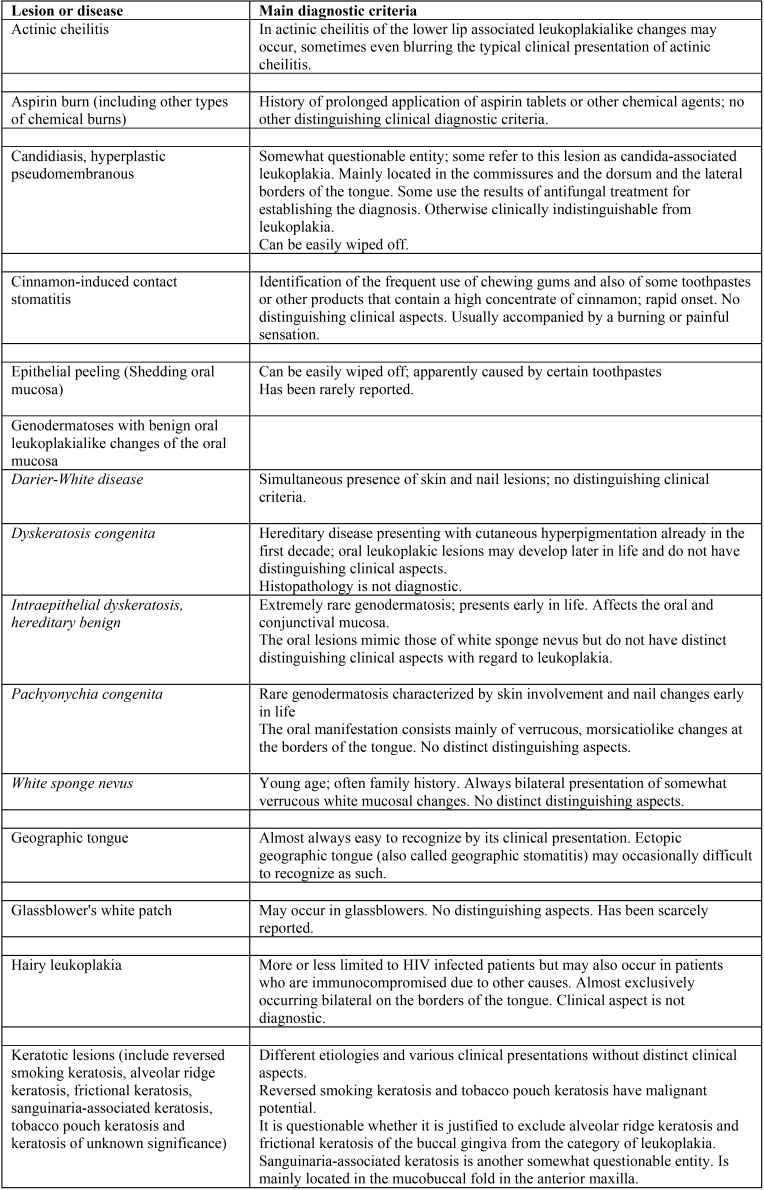
Well-defined, predominantly white lesions or diseases that should be excluded from a clinical diagnosis of oral leukoplakia.

**Table 2 continue:**
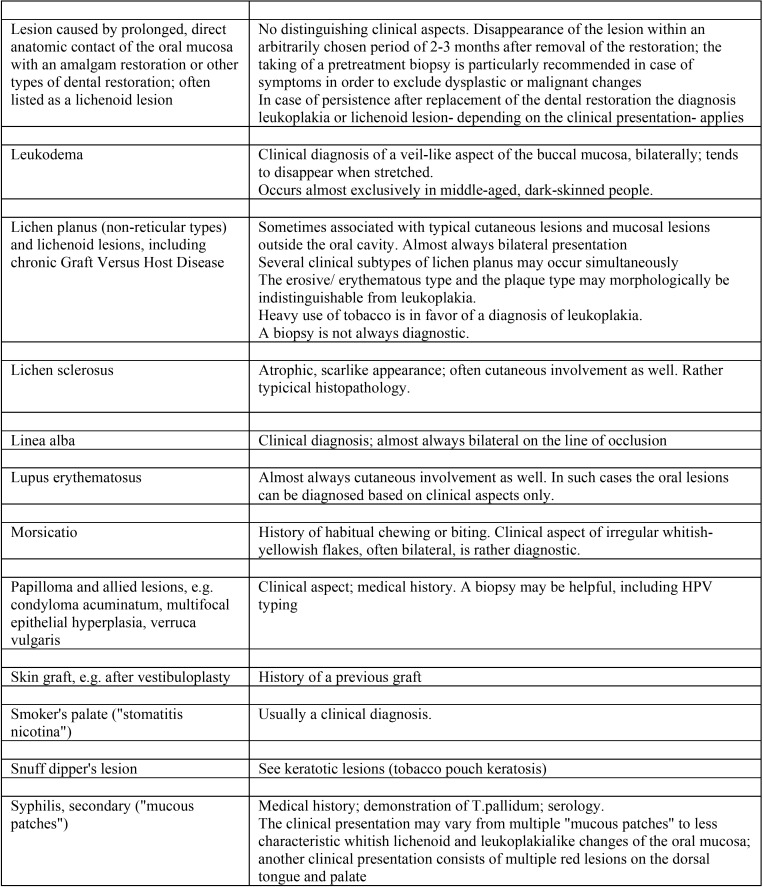
Well-defined, predominantly white lesions or diseases that should be excluded from a clinical diagnosis of oral leukoplakia.

The benign white oral mucosal lesions that may be encountered in genodermatoses should not be difficult to diagnose by the dentist, provided the patients have, indeed, disclosed their medical history. This partly also applies to the diagnosis of hairy leukoplakia although there are cases in which the patient is unaware of the underlying HIV infection or immunosuppressive state for other reasons. Another challenge is the diagnosis of white oral lesions as manifestation of the second stage of syphilis. Here, too, the patient may be unaware of the relevance of lifestyle aspects or may be reluctant to disclose these to the dentist. In such instances, a biopsy or another diagnostic aid, particularly serology, may be required.

In some of the listed entities it is questionable whether or not to consider them as a well-defined entity and not as leukoplakia. This particularly applies to “epithelial peeling” (3) and glassblower’s white patch; (4) alveolar ridge keratosis (5) and frictional keratosis of the buccal gingiva are other examples (6). Little is known about the possible potentially (pre)malignant character of Sanguinaria-associated keratosis and, therefore, it is actually unknown whether it is justified to separate this lesion from the category of leukoplakia (7). Yet another subject of debate is the Cinnamon-induced contact stomatitis (8).

In case of a possible etiologic factor, such as tobacco use or the presence of an amalgam restoration in close contact with the lesion, the dentist may await the result of the elimination of such factor for no longer than a somewhat arbitrarily chosen period of three months, provided the patient is asymptomatic otherwise. In the presence of symptoms a biopsy should be taken first. In case of a provisional diagnosis of cinnamon-induced lesions or Sanguinaria-associated keratosis it may be difficult to prove the causitive role of such agents.

In the absence of possible etiologic factors or lack of response to the elimination of such factors, referral to a specialist is recommended, both for assessment of the final diagnosis, the further managment of the patient and the provision of adequate patient information.

**Figure 1:**
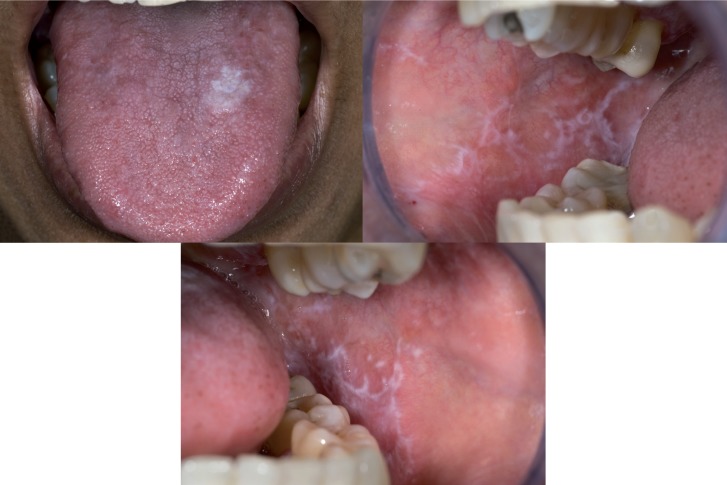
White lesion at the dorsum of the tongue that clinically qualifies for leukoplakia, plaque type lichen planus and perhaps also for hyperplastic candidiasis (a); only because of the simultaneous presence of lichen planus lesions elsewhere in the oral cavity there is a strong preference to diagnose the lingual lesion as plaque type lichen planus (b and c).

Probably the most common diagnostic challenge is the distinction between leukoplakia and non-reticular lichen planus and lichenoid lesions from. An example is shown in figure 1. Such distinction is of importance for the management of the patient, which partly relates to the issue of potential (pre)malignancy of leukoplakia and partly to the issue of treatment in case of symptoms, particularly when the use of topical steroids is considered. In such cases a biopsy may be helpful, but even then there may remain cases that can not be clearly diagnosed as either leukoplakia or lichen planus or lichenoid lesion.

As a general rule each leukoplakia should be biopsied irrespective of the presence or absence of symptoms, the clinical subtype (homogeneous or non-homogeneous), the seize and the oral subsite. In extensive leukoplakias the taking of multiple biopsies (“mapping”) may be considered. Probably in most parts of the world dentists are not trained to perform incisional or excisional biopsies; therefore, referral to a specialist for such procedure is advised.

The treatment policy, particularly in leukoplakias that are otherwise asymptomatic, is an issue of debate in view of the questionable effectiveness of whatever type of surgical or non-surgical treatment (1).

## Discussion

The present definition of leukoplakia has been worded in a negative way by excluding well-defined predominantly white lesions. Therefore, the accuracy of the clinicians’ diagnosis very much depends on their diagnostic capabilities. The use of artificial intelligence may become of great help in the near future to support the dentist in obtaining a correct clinical diagnosis of leukoplakia, similar as has been demonstrated in the clinical diagnosis of melanoma of the skin by the use of dermascopic pictures (9). Incorporated in smartphones artificial intelligence might then become a valuable instrument for use by dentists in the diagnostic process of predominantly white lesion of the oral mucosa.

The various terminologies that are being used in relation to the many predominantly white lesions of the oral mucosa, as shown in table II, are somewhat confusing, particularly at the level of the dentist-general practitioner. For instance, hairy leukoplakia is clearly a misnomer since 1) it is a well-defined entity, 2) it is not a potential (pre)malignant lesion, and 3) the lesion is not always “hairy” clinically. The category of so-called keratotic lesions is another source of confusion. Some of these lesions, such as reversed smoking keratosis and tobacco pouch keratosis are indisputily potential (pre)malignant and are not always characterized histopathologically by (hyper)keratosis. Admittedly, the risk of malignant transformation in predominantly white lesions of the alveolar ridge (“alveolar ridge keratosis”) and the buccal gingiva (“frictional keratosis”) is lower than for similar lesions located on the borders of the tongue or the floor of the mouth. It seems questionable, however, to remove such lesions from the category of leukoplakia. Of course, it is well recognized that avoidance of the term leukoplakia for lesions which carry a low risk of malignant transformation is an attractive option with regard to patient information.

The distinction between leukoplakia and non-reticular lichen planus seems to be the biggest challenge in the clinical diagnostic process. Theoretically, these lesions may occur simultaneously or may perhaps transform in time in one another, e.g. lichen planus transforming into leukoplakia.

The recommendation that each leukoplakia should be biopsied may be somewhat questionable at the specialists’ level, but seems a safe advise for dentists.

## Recommendation

In case of a clinical diagnosis of leukoplakia or where such diagnosis is part of the differential diagnosis the dentist-general practitioner is advised to look for consultation with a specialist both for confirmation or exclusion of the diagnosis and the further management of the patient. Also patient information about leukoplakia can probably better be provided by a specialist than by a general practitioner who is rarely confronted with this lesion.

## References

[1] Lodi G, Franchini R, Warnakulasuriya S, Varoni EM, Sardella A, Kerr AR (2016). Interventions for treating oral leukoplakia to prevent oral cancer. Cochrane Database Syst Rev.

[2] Warnakulasuriya S, Johnson NW, van der Waal I (2007). Nomenclature and classification of potentially malignant disorders of the oral mucosa. J Oral Pathol Med.

[3] Zegarelli DJ, Silvers DN (1994). Shedding oral mucosa. Cutis.

[4] Schiodt M, Larsen V, Bessermann M (1980). Oral findings in glassblowers. Community Dent Oral Epidemiol.

[5] Natarajan E, Woo SB (2008). Benign alveolar ridge keratosis (oral lichen simplex chronicus): A distinct clinicopathologic entity. J Am Acad Dermatol.

[6] Mignogna MD, Fortuna G, Leuci S, Adamo D, Siano M, Makary C (2011). Frictional keratoses on the facial attached gingiva are rare clinical findings and do not belong to the category of leukoplakia. J Oral Maxillofac Surg.

[7] Eversole LR, Eversole GM, Kopcik J (2000). Sanguinaria-associated oral leukoplakia: comparison with other benign and dysplastic leukoplakic lesions. Oral Surg Oral Med Oral Pathol Oral Radiol Endod.

[8] Allen CM, Blozis GG (1988). Oral mucosal reactions to cinnamon-flavored chewing gum. J Am Dent Assoc.

[9] Esteva A, Kuprel B, Novoa RA, Ko J, Swetter SM, Blau HM (2017). Dermatologist-level classification of skin cancer with deep neural networks. Nature.

